# The Prognostic Value of Autophagy-Related Markers Bclin-1 and LC-3 in Colorectal Cancers: A Systematic Review and Meta-analysis

**DOI:** 10.1155/2020/8475840

**Published:** 2020-03-23

**Authors:** Jin-xiao Li, Qian Yan, Na Liu, Wen-jiang Zheng, Man Hu, Zhao-min Yu, Yu-dian Zhou, Xiong-wen Wang, Feng-xia Liang, Rui Chen

**Affiliations:** ^1^Department of Integrated Traditional Chinese and Western Medicine, Union Hospital, Tongji Medical College Huazhong University of Science and Technology, 430022 Wuhan, China; ^2^Guangzhou University of Chinese Medicine, 510405 Guangzhou, China; ^3^Department of Acupuncture and Moxibustion, Chengdu University of Chinese Medicine, 610075 Chengdu, China; ^4^Department of Acupuncture and Moxibustion, Hubei University of Chinese Medicine, 430065 Wuhan, China; ^5^First Affiliated Hospital of Guangzhou University of Traditional Chinese Medicine, 510405 Guangzhou, China

## Abstract

**Objective:**

At present, the relationship between autophagosomes and the prognosis of various cancers has become a subject of active investigation. A series of studies have demonstrated the correlation between autophagy microtubule-associated protein light chain 3 (LC-3), Beclin-1, and colorectal cancer (CRC). Since autophagy has dual regulatory roles in tumors, the results of this correlation are also uncertain. Hence, we summarized the relationship between Beclin-1, LC-3, and CRC using systematic reviews and meta-analysis to clarify their prognostic significance in it.

**Methods:**

PubMed, EMBASE, Cochrane Library, and Web of Science databases were searched online up to April 1, 2019. The quality of the involving studies was assessed against the Newcastle-Ottawa Scale (NOS). Pooled hazard ratio (HR) and 95% confidence interval (CI) in a fixed or random effects model were used to assess the strength of correlation between Beclin-1, LC-3, and CRC.

**Results:**

A total of 9 articles were collected, involving 2,297 patients. Most literatures scored more than 6 points, suggesting that the quality of our including research was acceptable. Our finding suggested that the expression of Beclin-1 was not associated with overall survival (HR = 0.68, 95% CI (0.31–1.52), *P*=0.351). Nonetheless, LC-3 expression exerted significant impact on OS (HR = 0.51, 95% CI (0.35–0.74), *P* < 0.05). Subgroup analysis exhibited that Beclin-1 expression was associated with OS at TNM stage III (HR = 0.04, 95% CI = 0.02–0.08, *P* < 0.05), surgical treatment (HR = 1.53, 95% CI (1.15–2.02), *P*=0.003), and comprehensive treatment (HR = 0.27 95% CI (0.08–0.92), *P*=0.036), respectively. Similarly, the results showed the increased LC-3 expression in CRC was related to OS in multivariate analyses (HR = 0.44, 95% CI (0.34–0.57), *P* < 0.05), stages (HR = 0.51, 95% CI (0.35–0.74), *P* < 0.05), and comprehensive treatment (HR = 0.44, 95% CI (0.34–0.57), *P* < 0.05).

**Conclusions:**

Autophagy-related proteins of LC-3 might be an important marker of CRC progression. However, since the number of the original studies was limited, more well-designed, large-scale, high-quality studies are warranted to provide more convincing and reliable information.

## 1. Introduction

Colorectal cancer (CRC), with its mortality rate occupying the third place, represents the third most common malignancy in men and women worldwide [1]. It is estimated that by 2030, the number of newly diagnosed cases and cancer-related deaths of CRC will reach more than 2.2 million and 1.1 million, respectively. In the meantime, the global burden is expected to increase by 60% [2]. Despite the substantial development in the diagnosis and treatment of CRC, such as surgery and neoadjuvant and palliative care, the 5-year overall survival (OS) rate for CRC reportedly was still as low as 23.2% and the 5-year cumulative mortality rate stood at 71.3% [3, 4]. Hence, it is necessary to look for an effective biomarker to predict the prognosis of CRC.

Autophagy is a catabolic process. It maintains homeostasis by engaging in cellular waste disposal and intracellular recycling to eliminate excess or damaged proteins and organelles, invading microorganisms or by providing substrates for energy production and biosynthesis during stress [5]. As a survival and prodeath mechanism, it has been actively studied and was found to be intimately related to the occurrence of CRC, serving as targets for the cancer treatment protocols [6]. An increasing number of studies reported that autophagy plays an important role in the tumorigenesis [7], with dual effects on tumors [8]. However, the role of autophagy in cancer depends on the types, stages, and sites of the tumor [9]. Autophagy could promote the survival of tumor cells [10] and lead to the death of tumor cells [11]. Due to the close relations with clinical treatment of cancer, it can serve as a biomarker for the prediction of the prognosis of tumor [12]. Previous studies [13] revealed that although the specific mechanism of autophagy in CRC is not completely clear, it is considered to play a critical role in CRC. The identification of autophagy-related biomarkers will not only reveal new biomarkers in the diagnosis and treatment of CRC but also provide patients with personalized treatment options.

Beclin-1 is a protein that plays a significant role in autophagy. It interacts with a variety of cofactors to trigger the autophagy protein cascade. The dysfunction of Beclin-1 may lead to tumorigenesis, immune dysfunction, or liver and neurodegenerative diseases [14]. Several studies have shown that the expression pattern of Beclin-1 is positively or negatively correlated with tumors [14], including hepatocellular carcinoma [15], non-small-cell lung cancer [16], and epithelial ovarian cancer [17]. The LC-3 family contains three isoforms (LC-3A, LC-3B, and LC-3C) [18]. LC3 protein is the basic component of the inner and outer membrane of autophagy, so it can be used as a suitable marker in autophagy [19]. As far as we know, Beclin-1 [20] and LC-3 [21] are the most commonly used autophagy-related markers, which play a role in the autophagy of CRC-related cells. However, the relationship between autophagy and CRC remains controversial. The loss of autophagy-related protein Beclin-1 is associated with poor prognosis in CRC [22]. On the other hand, Koustas et al. [23] showed that the overexpression of Beclin-1 indicated a poor prognosis in CRC patients receiving chemotherapy. The high expression of LC-3 is positively correlated with the long-term survival of patients with CRC, which could be used as a biomarker for its prognosis [24]. Although increased LC-3 expression was mildly associated with poor prognosis, in the KRAS mutant group, LC-3 overexpression was significantly associated with decreased OS [25]. It is generally acknowledged that meta-analysis is a powerful statistic tool to overcome the limitation of different sample sizes from individual studies and to generate the best estimation. Hence, we conducted this meta-analysis to investigate the relationship between Beclin-1, LC-3, and the OS of CRC.

## 2. Materials and Methods

### 2.1. Literature Search Strategy

Four major English databases, including PubMed, EMBASE, Cochrane Library, and Web of Science, were searched online. In addition, the literature of Beclin-1 and LC-3 related to the prognosis of CRC were searched too. The time frame of search ranged from the past to April 1, 2019. Search terms included “Beclin-1,” “Beclin-1 Protein,” “GT197 Protein,” “ATG-6 Protein,” “ATG Protein 6,” “Coiled-coil Myosin-like Bcl2-interacting Protein,” “Coiled coil Myosin-like Bcl2 interacting Protein,” “Beclin1,” “ATG6 Protein,” “LC-3 Protein, human,” “microtubule-associated Protein 1 light chain 3,” “Microtubule-associated protein 1 light chain 3,” “LC-3 protein, human,” “microtubule-associated protein light chain 3,” “light chain 3,”“LC-3,” “Neoplasms, Colorectal,” “Colorectal Neoplasm,” “Neoplasm, Colorectal,” “Colorectal Tumors,” “Colorectal Tumor,” “Tumor, Colorectal,” “Tumors, Colorectal,” “Colorectal Carcinoma,” “Carcinoma, Colorectal,” “Carcinomas, Colorectal,” “Colorectal Carcinomas,” “Colorectal Cancer,” “Cancers, Colorectal,” and “Colorectal Cancers.” We combined subject headings with key words in our search strategy. At the same time, we also manually searched the list of references for relevant reviews to find eligible studies that might have been missing, including articles and related reviews.

### 2.2. Selection Criteria

The inclusion criteria were as follows: (1) the diagnosis for patients was made against pathological criteria; (2) studies investigated the relationship between the prognosis of patients with CRC and LC-3, Beclin-1; (3) provided sufficient information to allow the estimation of the OS; and (4) published in English. Furthermore, studies will be rejected if they satisfy any of the following criteria: (1) experiments on animals and cells; (2) meta-analysis, letters, or reviews; (3) unavailability of full-text versions or incomplete data; and (4) irrelevant to the subject. For multiple or redundant publications from the same population, only the most recent or most complete studies were included in the analysis.

### 2.3. Data Extraction and Quality Assessment

All original studies retrieved were evaluated and screened independently by two authors (Jin-xiao Li and Qian Yan). Any disagreement about the eligibility of an article between the two searchers was determined by a third author (Rui Chen), who would determine whether an article would be included or not. Each study included the following basic information: the first author, year of publication, country, median age, gender, tumor site, the total number of cases, TNM staging, grade, treatment strategy, detection method, and follow-up time. The quality of our research was assessed against the Newcastle-Ottawa Scale (NOS) [26]. The NOS consists of three parts: study population selection (0–4 points), comparability between groups (0–2 points), and the results of measurement (0–3 points). Studies with a score of six on the scale are deemed to be of high-quality.

### 2.4. Statistical Analysis

The STATA (Version: 12.0 College Station, TX) software was used for all statistical analysis. The required HRs and 95% CIs were extracted for survival analysis. Most of the literature directly provided such data, and for some studies, it had to be extracted through the K-M survival curve [27]. For the value of HR in both univariate and multivariate analyses, we chose to retain the HR and 95% CI of the multivariate analyses and then integrated them into a subgroup analysis of the univariate and multivariate analyses. Cochran's *Q* test and Higgins I-squared statistic were undertaken to assess the heterogeneity of the included trials. If *P* > 0.10 or *I*^2^ < 50%, it indicates homogeneity, and then the fixed effect model was taken to further calculate. Otherwise, the random effect model was used. Then, subgroup analysis and sensitivity analysis were conducted to explore the sources of heterogeneity and analyze them. Egg's funnel plot was drawn to determine whether publication bias existed in the included studies [28]. Harbord-weighted linear regression was used to test publication bias quantitatively. When *P* < 0.05, the publication bias was considered to be statistically significant.

## 3. Results

### 3.1. Study Selection and Characteristics

**Figure 1:**
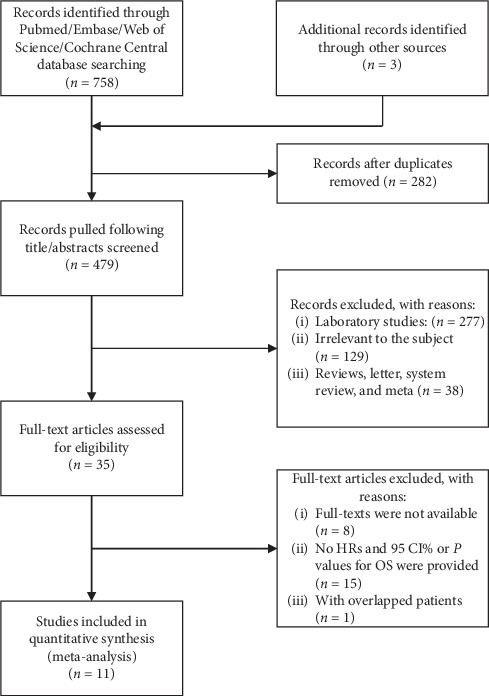
Flow diagram of study selection.

A total of 479 relevant studies were selected for screening after removing 282 duplicated records from 758 studies. The flow chart is detailed in Figure 1. In the screening process, the title and abstract of relevant articles were read by two authors (Jin-xiao Li and Qian Yan) independently, and 444 citations were excluded after the first screening, with 26 included for full-text review. After meticulous evaluation, only nine studies were identified to satisfy the inclusion criteria for further analysis [22, 24, 25, 29–34], including eight about “Beclin-1 and CRC” [24, 25, 29–34] and four about “LC-3 and CRC” [22, 24, 31, 32].

### 3.2. Methodological Quality of Selected Studies

Table 1 lists the major characteristics of the nine included studies. All studies employed immunohistochemical (IHC) staining in formalin-fixed paraffin-embedded tissues for the detection of Beclin-1 and LC-3. The methodological score of each study on the NOS scale was provided in Table 1. Studies scoring 1–3, 4–6, and 7–9 on the NOS scale were listed as low-, intermediate-, and high-quality ones, respectively. The overall mean median of the included studies was seven points (five–seven points), indicating that the quality of the original studies included was reasonable. Eight studies examined the association between the Beclin-1 expression and OS, and four studied the correlation between the expression of LC-3 and it.

### 3.3. Correlation between LC-3 Overexpression and Increased OS in CRC Subjects

**Figure 2:**
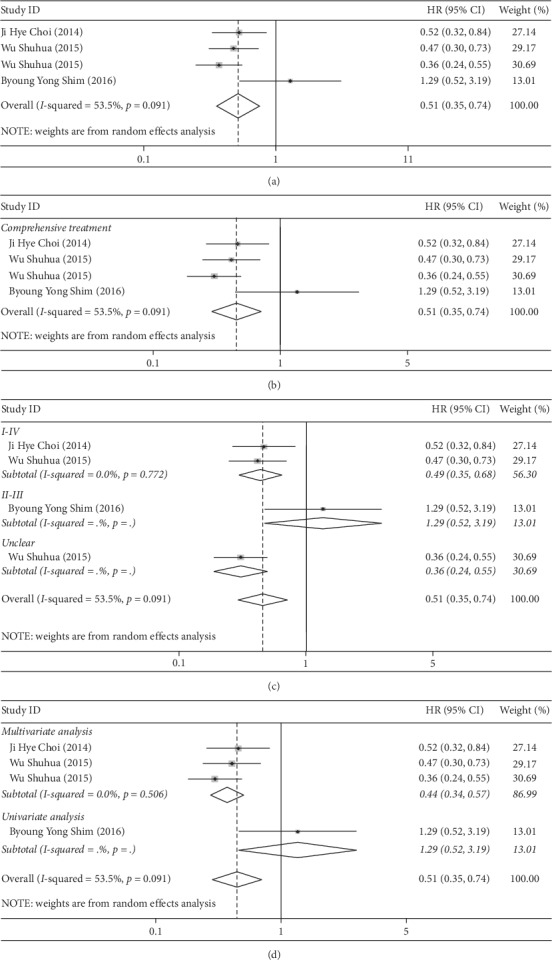
(a). Forest plot of hazard ratio (HR) for the association between LC-3 and OS; forest plot of subgroup analysis of treatment (b), stage (c), and variable type (d) with OS.

A total of 885 patients with CRC from four trials were analyzed to assess the prognostic value of LC-3 in CRC. The heterogeneity test was performed first. Since *I*^2^ = 53.5% and *P* = 0.091 suggest heterogeneity, the random effect model was used (Figure 2(a)). In the follow-up analysis, we conducted subgroup analysis and sensitivity analysis. The results showed that increased expression of LC-3 was a protective factor for OS (HR = 0.51, 95% CI (0.35–0.74), *P* < 0.001). At the same time, subgroup analysis was conducted in terms of treatment modalities, stages, and univariate and multivariate analysis. This analysis revealed that all patients were treated with comprehensive therapy, and the overexpression of LC-3 was a protective factor for OS in treatment (HR = 0.51, 95% CI (0.35–0.74), *P* < 0.001) (Figure 2(b)). As is shown in Figure 2(c), increased LC-3 expression in patients with stage I-IV CRC was a protective factor for OS (HR = 0.49, 95% CI (0.35–0.68), *P* < 0.001), as well as in multivariate analysis (HR = 0.44, 95% CI (0.34–0.57), *P* < 0.001). Yet, the univariate analyses revealed that increased LC-3 was not associated with OS (Figure 2(d)).

### 3.4. Correlation between Beclin-1 Overexpression and Increased OS in CRC Subjects

**Figure 3:**
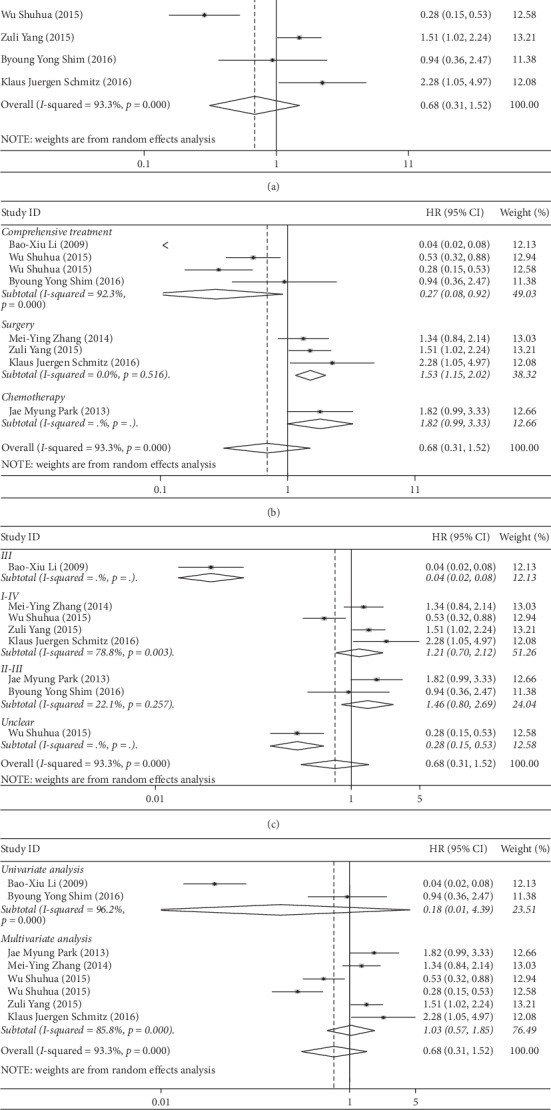
(a). Forest plot of hazard ratio (HR) for the association between Beclin-1 and OS; forest plot of subgroup analysis of treatment (b), stage (c), and variable type (d) with OS.

First, the heterogeneity test was performed. As shown in Figure 3(a), *I*^2^ = 93.3%, and *P* < 0.001, there was a slight degree of heterogeneity existing in our study. Hence, the random effect model was adopted to analysis. The results showed that there existed no correlation between the increased expression of Beclin-1 and OS (HR = 0.68, 95% CI (0.31–1.52), *P*=0.351). To evaluate the source of heterogeneity, subgroup analysis was carried out in light of treatment modalities, stages, single, and multifactors. Our study showed that the increase of Beclin-1 was a protective factor for OS (HR = 0.27, 95% CI (0.08–0.92), *P*=0.036) in subjects treated by comprehensive treatments (Figure 3(b)). Elevated Beclin-1 expression was a risk factor for OS in surgically-treated patients (HR = 1.53, 95% CI (1.15–2.02), *P*=0.003). The expression of Beclin-1 in patients treated with chemotherapy alone was independent of OS (HR = 1.82,95% CI (0.99–3.33), *P*=0.052). As shown in Figure 3(c), upregulated Beclin-1 expression in stage III CRC patients was a protective factor for OS (HR = 0.04, 95% CI (0.02–0.08), *P* < 0.001). However, the increased Beclin-1 expression bore no relationship with the improved OS in the univariate and multivariate analysis (Figure 3(d)).

### 3.5. Sensitivity Analysis and Publication Bias

**Figure 4:**
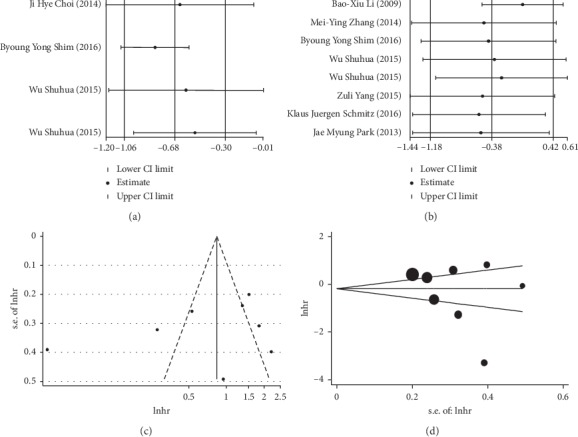
Sensitivity analysis of the correlation between LC-3 (a) and Beclin-1 (b) expression and overall survival; funnel plot for the assessment of publication bias for Beclin-1 (c, d).

The correlation between the expression of LC-3 (*I*^2^ = 53.5%, *P* = 0.091), Beclin-1(*I*^2^ = 93.3%, *P* < 0.001) and OS were significantly heterogeneous among the studies included. In order to further assess the sources of heterogeneity, we used the leave-one-out sensitivity analyses by removing one study per time to check if individual study influenced the results. Since no substantial change was found, the sensitivity analysis of LC-3 (Figure 4(a)) and Beclin-1 (Figure 4(b)) showed that no individual study affected the pooled results.

Furthermore, the funnel plot and Begg's test were performed to estimate the publication bias of the included studies. In terms of the correlation between CRC prognosis and LC-3, the funnel plot and Begg's test revealed no significant publication bias for OS (*P* = 0.089). As to the correlation between the CRC prognosis and Beclin-1, no evidence of publication bias was observed (*P* = 0.266) (Figures 4(c) and 4(d)). The reason for no significant publication bias may be that fewer articles were included.

## 4. Discussion

The roles that autophagy plays in tumorigenesis have been actively investigated for years, yet researchers still failed to reach a consensus on the relationship between autophagy and cancer. Autophagy has been suggested to play a dual role in carcinogenesis. On the one hand, it suppresses tumor development by preventing the accumulation of redundant intracellular molecules that may generate toxic products favoring genomic instability and thus adding to neoplastic transformation [35]. On the other hand, autophagy promotes tumor growth under stress conditions, such as hypoxia, starvation, or presence of reactive oxygen species (ROS), leading to the increase of survival index by preventing apoptosis. [36] Therefore, despite the fact that autophagy protects normal cells against neoplastic transformation, this process also endows tumor cells with a mechanism that enables their survival under stress/adverse conditions [37]. In view of this, we reviewed the published clinical studies and undertook a meta-analysis with an attempt to assess the prognostic value of Beclin-1 and LC-3 in CRC in our study. To our knowledge, this meta-analysis is the first of its kind to examine the association between Beclin1/LC-3 and clinical features and prognosis of CRC.

Overall, our meta-analysis pooled the outcomes of 2297 patients with CRC from 9 individual studies and found that the expression of Beclin-1 was not associated with OS (HR = 0.68, 95% CI 0.31–0.52, *P* > 0.05). Subgroup analysis showed that the expressions of both Beclin-1 (HR = 0.27, 95% CI 0.08–0.92) and LC-3 (HR = 0.51, 95% CI 0.35–0.74) were associated with treatment modalities (surgery and chemotherapy versus comprehensive treatment). We found that Beclin-1 overexpression was associated with reduced survival in the surgically-treated CRC patients, while in the patients treated with comprehensive treatment, elevated Beclin-1 levels was associated with prolonged OS. Studies have shown that reduced expression of Beclin-1 can inhibit autophagy activity and proliferation and promote apoptosis of CRC cells [20]. Upregulating the expression level of Beclin-1 can increase the autophagy of CRC cells, thereby antagonizing cetuximab-induced cell death [38]. Consistent with these findings, an increased level of Beclin-1 expression was strongly associated with longer 5-year OS in patients with locally advanced CRC who were treated with 5-FU chemotherapy for six months after surgery [29]. Besides, our findings indicated that overexpression of Beclin-1 might be a protective factor in CRC patients with stage III, which was also consistent with the findings of others studies [29, 39]. High Beclin-1 expression has been linked to a good prognosis and longer OS in CRC patients with stage IIIB [33].

Another important protein involved in the autophagy is LC-3, the content of which is related to the level of autophagy [40, 41]. Our results indicated that the elevated expression of LC-3 might be a protective factor for the OS of CRC patients. Specifically, the increased LC-3 expression in CRC patients who were treated with comprehensive therapy or in those at stage I-IV was a protective factor for OS. Meanwhile, the multivariate analysis yielded similar results. LC-3 might serve as a marker for prognostic evaluation and a novel target for CRC therapy. Comprehensive therapeutic approaches have great potential in the treatment of colorectal tumors [42]. However, LC-3 overexpression was reportedly correlated with poor prognosis of patients with breast, ovarian, and lung carcinomas [43–45]. The most likely explanation is that autophagy plays a dynamic role in cancer. It has both antitumor and tumorigenic effects, which depends on a variety of factors, including tumor stage, cellular context, tissue of origin, and so on [46].

We analyzed the heterogeneity of this study and found slight heterogeneity in the research of Beclin-1 (*I*^2^ = 93.3%, and *P* < 0.001) and LC-3 (*I*^2^ = 53.5% and *P*=0.091). Then, we performed subgroup analysis and sensitivity analysis and did not find the root cause of heterogeneity. One the one hand, the heterogeneity of this study may come from the imbalance of the clinical factors. However, since the subgroup analysis of the treatment methods, TNM staging, univariate, and multivariate analysis did not find the source of heterogeneity, we considered that the clinical heterogeneity of our study may be derived from sex ratio, pathological grade, tumor size, and number of samples. On the other side, the limited number of our studies may also be part of the heterogeneity source. In addition, according to the results of the Begg's test, there was no publication bias from in our meta-analyses of the associations of LC-3 (*P*=0.089) or Beclin-1 (*P*=0.266) with the OS in CRC patients. Since the Begg's method also originates from a visual evaluation of the funnel plot, when the number of studies included is small, the efficiency of the method is relatively reduced. Therefore, it is consistent with the results observed in the funnel graph, namely, that there may be some publication bias in this article.

To date, our current meta-analysis is the first to evaluate the relationship between Beclin-1, LC-3 and OS in CRC patients. Meanwhile, the latest comprehensive data was collected in this article, and we made a detailed analysis of the staging and treatment of CRC. However, several limitations did exist in our study. First, all the studies used a retrospective design that has inherent limitations. We cannot get complete clinical and pathological information, and the follow-up time was limited. Hence, the data of progression-free survival (PFS), disease-specific survival (DSS) and disease-free survival (DFS) of patients was lacking, which are of great significance for patients with CRC. On the other side, the value of OS was extracted from K-M plotter rather than from original data of variance in some studies, which may affect the estimation of potential interactions.

## 5. Conclusion

To sum up, this systematic review and meta-analysis indicated that elevated expression of LC-3 predicted a favourable OS in CRC patients, whereas the expression of Beclin-1 was not associated with it. Although the clinical application of these autophagy-related markers is still waiting for further confirmation, this is the first study to comprehensively analyse the correlation between autophagy-related proteins Beclin-1, LC-3 and OS of CRC patients, showing a certain prognostic value. We believe that autophagy-related prognostic proteins will be more and more widely used in CRC.

## Figures and Tables

**Table 1:** Features of trials included.

Year	Lead author	Country	Median age	TNM stage (I/II/III/IV)	Tumor site (rectum/colon)	Grade (I/II/III)	Method	Sample size (M/W)	Treatment	Survival analysis	Marker	Follow-up period	NOS
2009	Li et al. [29]	China	60	IIIb	Left hemicolon (70)/right hemicolon (45)	6/86/23	IHC	115 (69/46)	Chemotherapy after operation	OS	Beclin-1	≥5 year	7

2013	Park et al. [30]	USA	63.5 (26–81)	II (32)/III (146)	Unclear	I/II (119)/III/IV (59)	IHC	178 (99/79)	Chemotherapy	OS	Beclin-1, LC-3	Unclear	5

2014	Choi et al. [22]	Korea	64 (30–83)	38/67/101/57	111/152	I/II (222)/III (41)	IHC	263 (141/122)	Surgery + concurrent chemoradiation therapy/chemotherapy	OS	Beclin-1, LC-3	71.4 (0.5–197.4) months	7

2014	Zhang et al. [34]	China	65	I/I (243)/III/IV (234)	Colon	260/269/35	IHC	589 (343/246)	Surgery	OS	Beclin-1	13–84 months	7

2015	Shu-hua et al. [32]	China	60	I/II (204)/III/IV (38)	115/164	80/139/60	IHC	279 (146/133)	Chemotherapy, operation	OS	Beclin-1, LC-3	10–89 months	7

2015	Shu-hua et al. [24]	China	60	Unclear	96/146	67/127/48	IHC	242 (127/115)	Chemotherapy, operation	OS	Beclin-1, LC-3	≥5 year	7

2015	Yang et al. [33]	China	60	52/152/121/38	173/190	31/289/43	IHC	363 (199/164)	Surgery	OS, DFS	Beclin-1	Unclear	7

2016	Shim et al. [31]	Korea	62	II/III	Colon	18/76/7	IHC	101(69/32)	Neoadjuvant chemoradiotherapy and laparoscopic TME	OS, RFS	Beclin-1, LC-3	≥5 year	5

2016	Schmitz et al. [25]	Germany	Unclear	I/II/IV/IV	80/47	28/69/19	IHC	127 (66/61)	Surgery	OS	beclin1, LC-3, p65	60 months	7
